# Editorial: The role of lipids in abiotic stress responses

**DOI:** 10.3389/fpls.2024.1378485

**Published:** 2024-03-06

**Authors:** Saroj Kumar Sah, Adriano Sofo

**Affiliations:** ^1^ Biology Department, Brookhaven National Laboratory, Upton, NY, United States; ^2^ Department of Natural and Applied Sciences, Nexus Institute of Research and Innovation (NIRI), Lalitpur, Nepal; ^3^ Department of European and Mediterranean Cultures, University of Basilicata Matera, Matera, Italy

**Keywords:** lipids, drought stress, heat stress, high temperature, lipid modulation, lipid remodeling

Lipids are one of the most important components of biological membranes including plasma membranes, which is the interface between the cell and the environment (Houe et al., 2016). The intricate relationship between lipids and plant responses to abiotic stress has garnered increasing attention in recent years. The role of lipids in abiotic stress response is complex and finely tuned interplay of membrane dynamics, signal transduction, and the synthesis of protective compounds. Unraveling the intricacies of lipid-mediated stress adaptation is essential for developing strategies to enhance the resilience of crops in the face of changing environmental conditions. Further research into lipidomics and lipid signaling pathways holds the promise of unlocking novel approaches for engineering stress tolerant crops, ensuring food security in a rapidly changing world. This Research Topic delves into the multifaceted role of lipids in plants’ ability to withstand and adapt to various environmental stressors such as drought and extreme temperatures. Understanding the intricate lipid-based mechanisms that contribute to stress response is crucial for developing resilient and stress-tolerant crops.

In this Research Topic, 5 articles have been put together, which comprise of original research as well as review articles. To advance a context on the theme and to lay a sturdy footing for the Research Topic, one review article has been published. The review paper entitled “*Drought and heat stress mediated activation of lipid signaling in plants: a critical review*” by Sharma et al., explores the role of lipids in signaling pathways that plants employ to cope with adverse environmental conditions. The authors emphasize the importance of understanding how lipid signaling is activated during drought and heat stress, shedding light on the molecular mechanisms, and signaling cascades involved. The review likely delves into lipid alterations, signal transduction pathways, and protective compounds derived from lipids as key components of plant stress response strategies. The drought affects the efficiency of water channels by increasing saturated fatty acyl chains and causing lipid peroxidation. Membrane fluidization occurs at high temperatures whereas membrane rigidification occurs at low temperature which leads to an increase in the saturation levels of membrane lipids and long hydrophobic fatty acid chain. Overall, the paper provides valuable insights into the complex interplay between lipids and stress adaptation in plants, contributing to our understanding of plant resilience in challenging environmental conditions.

One imperative area in the field of abiotic stress mitigation is drought stress, thus, a better understanding of drought adaptation and tolerance mechanisms is crucial for the breeding of more drought tolerant lines, therefore, a research paper entitled “*Lipid remodeling of contrasting maize (Zea mays L.) hybrids under repeated drought*” by Kränzlein et al., was also included in this Research Topic. In this research, the authors examined two maize hybrids (i.e. K and L) with comparable growth, however, have contrasting drought responses were investigated using different physiological, metabolic and lipidomic tools to understand the plant strategies of lipid remodeling in response to repeated drought stress response. Results indicate significant alterations in lipid composition under drought stress, with variations observed between the different hybrids. These findings contribute to understanding the adaptive mechanisms of maize plants to drought stress at the lipid level, which could potentially aid in breeding more resilient varieties.

Likewise, another stress is high temperature which impacts the lipid biosynthesis and ultimately impacts on yield loss. Understanding the physiological and genetic foundations of heat tolerance provides the groundwork for creating tools to breed different crop varieties resilient to high temperatures. At the cellular level, damage to membrane is a primary factor leading to yield loss during heat stress (Ernst et al., 2016). Many crops’ yields are affected due to high temperature, for example peanuts. Peanuts ranks among the top 10 food crops worldwide in terms of harvested area (Food and Agriculture Organizations of the United Nations FAOSTAT statistical database, 2022). Peanuts serve as an affordable source of protein, fatty acids, antioxidants, and vitamins. They play a crucial role in addressing both malnutrition and obesity, offering nutrition to the undernourished while aiding in managing weight in the overnourished (Higgs, 2003). This makes peanuts a vital crop for combating hunger and malnutrition, both domestically in the United States and internationally. In the United States alone, approximately 2885 thousands metric tons were produced during the 2021-2022 production season, positioning the United States as the fourth largest producer of peanuts globally (United States Department of Agriculture-Foreign Agricultural Service, 2023). Peanuts are severely affected by harsh environments such as drought and high temperatures (Puppala et al., 2023). Therefore, to understand the role of temperature in lipid modulation the paper entitled “*Lipid modulation contributes to heat stress adaptation in peanut*” by Spivey et al., was included in this Research Topic. They studied the peanut lipidome in leaf tissues. They showed that at high temperatures, significant changes in lipid composition occur which involves decrease in unsaturation levels primarily through reduction of 18:3 fatty acid chains within both plastidic and extra-plastidic diacyl membrane lipids. However, the levels of triacylglycerols (TGs) containing 18:3 increase at high temperature, indicating a potential role for TGs in sequestering fatty acids during membrane lipid remodeling under plant stress. Their findings propose that the transfer of 18:3 chains from membrane diacyl lipids to TGs and sterol esters (SEs) is a crucial aspect of lipid remodeling for high temperature adaptation in peanuts leaves. Additionally, the application of QTL-seq enabled the identification of a genomic region associated with heat-adaptive lipid remodeling, offering insights for the identification of molecular markers linked to heat tolerance.

Suboptimal and excessive temperature have adverse effects on both growth and photosynthetic efficiency, influencing subsequent lipid accumulation. Lower temperature triggers an increase in fatty acid desaturation while higher temperature triggers the opposite reaction. The effect of temperature on lipid classes has been studied less in microalgae. The research paper entitled “*Growth and fatty acid distribution over lipid classes in Nannochloropsis oceanica acclimated to different temperatures*” by Ferrer-Ledo et al., studied the effect of temperature on growth, photosynthesis, and lipid class accumulation in microalgae. The study showed that lower temperature leads to a decrease in photosynthesis rates and an increase in diacylglyceryltrimethylhomoserine (DGTS) content. Lower temperature triggered a profound remodeling of lipid classes, especially the proportion and fatty acid composition of polar lipids. The increase in DGTS content suggests that a role of this lipid class in low temperature tolerance. Also, triacylglycerol content increased at 17°C and decreased at 9°C emphasizing a metabolic switch in stress response.

Very long chain fatty acids (VLCFAs) are fatty acids characterized by chain lengths of 20 or more carbon atoms. VLCFAs are crucial constituents of various lipid classes found in wide range of organisms, from bacteria to higher plants and animals, including humans (Kyselová et al., 2022). VLCFAs are important molecules that play an important role in physiological and structural roles in plants and their derivates play in the interaction of plants with their abiotic and biotic environment (Batsale et al., 2021). The synthesis of VLCFAs is primarily controlled by 3-ketoacyl-CoA synthase (KCS). This enzyme is considered the key rate-limiting step in the biosynthesis of VLCFAs. The KCS gene family is not well studied in soybean so far. Therefore, another research paper entitled “*Genome-wide identification and expression analysis of the KCS gene family in soybean (Glycine max) reveal their potential roles in response to abiotic stress*” by Gong et al., described the comprehensive study that explored the distribution, classification, and expression profiles of GmKCS genes, shedding light on their potential role in developmental processes and stress responses in soybean. In this study, 31 KCS genes were identified in the soybean genome which are distributed unevenly on 14 different chromosomes. The differential expression of GmKCS genes in soybean leaves under these stress conditions implies their potential involvement in soybean resistance to environmental challenges. The inclusion of cis-acting element analysis, which indicates the presence of hormones- and stress-responsive elements in GmKCS promoters, further supports the idea that the expression of these genes may be regulated by various developmental and environmental stimuli. Overall, this research provides fundamental information about the soybean KCS gene family, laying the groundwork for future functional elucidation and exploitation of these genes in enhancing soybean resilience to abiotic stress.

In conclusion, abiotic stresses still pose a great threat to sustainable agricultural production. Current changing climate conditions will become more threat for crop growth and productivity. Understanding the intricate interplay of lipids in these processes is essential for developing strategies to enhance plant resilience to abiotic stress, ultimately contributing to the sustainable production of crops in the challenging environmental conditions.

## Author contributions

SS: Conceptualization, Writing – original draft, Writing – review & editing. AS: Writing – review & editing.
